# The Transition to Kindergarten for Hispanic and Latine Autistic Children: A Focus Group Study with Caregivers

**DOI:** 10.1007/s10803-025-06721-2

**Published:** 2025-01-22

**Authors:** Emily Jellinek-Russo, Milena Keller-Margulis, Sarah S. Mire, Ivana Lozano, Brenda Duran, Rachel H. Fein, Jorge Gonzalez, Susan X. Day

**Affiliations:** 1 Child Development Center, Department of Pediatrics, Nationwide Children’s Hospital, Columbus, OH USA; 2 Department of Psychological, Health, & Learning Sciences, University of Houston, Houston, TX USA; 3 Department of Educational Psychology, Baylor University, Waco, TX USA; 4 Legacy Community Health, Houston, TX USA

**Keywords:** Kindergarten, Transition, Autism, Caregivers

## Abstract

*Purpose*: Past research highlights the different facilitators and barriers that caregivers of children on the autism spectrum experience during the transition to kindergarten and when navigating special education services. Caregivers who identify as Hispanic and/or Latine may face distinct challenges during this process, such as language differences, differences in understanding autism and special education, and barriers to advocating for their child. Hispanic and Latine caregivers also have strengths, resources, and strategies (i.e. cultural capital) that they use during this time. However, there is little research aimed at understanding the unique experiences of Hispanic and Latine caregivers of autistic children during their entry to kindergarten. *Methods*: To address this shortcoming, the current study used qualitative methods and thematic analysis to explore the transition to kindergarten experiences of four caregivers of autistic children. *Results*: This study identified strengths, supportive practices, and challenges that participants experienced fell under four major themes: importance of proactive and ongoing partnerships between caregivers and schools, navigating unfamiliar language and processes, the need for dissemination of information about autism to teachers and support from trusted systems. Themes highlighted challenges such as communication differences, unfamiliar school processes, community and teacher misconceptions about autism. Facilitators the transition included proactive communication, shared goals and partnerships with school. Yosso’s Community Cultural Wealth Framework is integrated into the discussion of themes and the forms of cultural capital participants used to support their child. *Conclusion*: Recommendations for practice and research to support Hispanic and Latine autistic children during the kindergarten transition are provided.

Beginning kindergarten is an important transition in a child’s life and influences future school success (McIntyre et al., 2006). However, many students experience challenges transitioning to the new environment. In a study of 688 kindergartners in the Midwestern United States, Jiang and colleagues (2021) found that more than 70% of children with and without disabilities had difficulty transitioning to kindergarten, including learning to make friends, follow schedules, meet academic demands, work within groups, and stay organized. Children with disabilities are even more likely to experience difficulties when starting kindergarten (Jiang et al., 2021). These findings suggest that for children in general, there is a mismatch between the expectations of kindergarten and children’s skill sets when they enter kindergarten.

The kindergarten transition may require additional and/or individualized supports from key stakeholders to facilitate the shift from preschool to kindergarten for autistic children, who may have different needs (e.g., communication, behavioral, adaptive) and fewer school readiness skills than non-autistic peers (Forest et al., 2004). Specifically, caregivers of autistic children transitioning into kindergarten must advocate for their children’s needs through communication and collaboration with key stakeholders, all while navigating the new personnel, procedures, and policies that typically come with moving into the formal education setting of kindergarten (Wildenger & McIntyre, 2011). These challenges in transition may be exacerbated when caregivers are from marginalized groups.

## Transition Experiences of Hispanic and Latine Caregivers of Autistic Children

The Hispanic and Latine community is the largest ethnic minority in the United States (U.S.) and is continuing to grow, accounting for 19% of the population (U.S. Census Bureau, 2020). As of 2020, 25% of U.S. children identified as Hispanic or Latine (U.S. Census Bureau, 2020). Within these communities lies great diversity across the culture, ancestry, and heritage of individuals. The term *Hispanic* has been used in the United States to refer to individuals who trace their origin from Spain and other Spanish speaking countries (e.g., Cuba, Central America, Mexico, Puerto Rico, South America). *Latine* and *Latinx* are nonbinary terms used in the United States to describe individuals who trace their ancestry to Latin America (Cuba, Mexico, Puerto Rico, and some countries in Central America, South America, and the Caribbean) (Atkin et al., 2022; Flanagin et al., 2021). While both are gender neutral terms, Latine may be more preferred by those who trace their ancestry to Latin America given its adherence to Spanish grammatical rules and common use by Spanish speakers (Mendez, 2023).

Hispanic and Latine children are twice as likely to have transition challenges (e.g., social difficulties, challenges following schedules and routine, adjustment to academic demands of kindergarten) compared to non-Hispanic children (Jiang et al., 2021). Given the increased challenges Hispanic and Latine children *and* autistic children face during the kindergarten transition, Hispanic and Latine children who have autism are likely to require extra support during this time (Jiang et al., 2021). Hispanic and Latine individuals are more likely to face delays in autism diagnosis and lower access to autism resources, interventions, and special education services compared to those who identify with the racial majority (Liu et al., 2023; Smith et al., 2020). Hispanic and Latine caregivers of autistic children often face more barriers advocating for their child and developing the school and home relationship compared to non-Hispanic and Latine caregivers. Various factors limit access to resources needed to develop and maintain relationships, including language proficiency, socioeconomic status, discrimination, power differentials between family and school staff, knowledge of special education (Burke et al., 2020, 2021; Liu et al., 2023; Smith et al., 2020).

Differences in values may also influence the family-school relationship during the kindergarten transition. Wong and Hughes (2006) found Latine families reported lower levels of shared education responsibility with their child’s teachers. Other research suggests the cultural values of respect (*respeto*) toward teachers may result in caregivers feeling apprehensive to communicate with and ask questions of teachers (Hoffman et al., 2020). Other cultural aspects may serve as facilitators during this process. For example, *(confianza)* the value of trust or confidence in someone/something, has been cited as an important value related to supporting parental engagement in educational contexts (Ijalba, 2015). Familism (*familismo*) represents a commitment to advocating for, respecting, and supporting family members within Hispanic and Latine communities. These values may drive caregiver practices during the kindergarten transition such as seeking out resources, communicating with schools, and advocating for their child (Burke et al., 2019). Further, Hispanic and Latine families may rely on religion, family, and other Hispanic/Latine families for social support and guidance navigating the educational system (Burke et al., 2019; Salkas et al., 2016).

To our knowledge, only one existing study examined transition experiences of culturally and linguistically diverse caregivers and their autistic children in the United States. Smith and colleagues (2021) conducted focus groups and individual interviews with 45 racially, linguistically, and/or economically minoritized caregivers of autistic children about their child’s school transitions, which broadly included the transition to “primary” and “secondary” school. Smith and colleagues (2021) found school administrators expected caregivers to “just know” what to do, as options related to services (e.g., transportation, where to access hot meals for their child over the summer, summer schooling) and other information about transition was not communicated to many caregivers. Although the caregivers in Smith et al. (2021) endorsed distinct challenges, findings underscore participants’ resourcefulness and determination in advocating for their children through aspects of cultural capital including accessing individualized transition plans, creating transition binders for students, and using services that increased caregiver engagement (e.g., shared calendars for meetings, informational posters and handouts, and consistent communication with school). Caregivers found other informal facilitators helpful to the transition process, including having positive relationships with teachers and other school staff (Smith et al., 2021). Notably, Spanish speaking, Hispanic/Latine caregivers reported transition facilitators such as access to community agencies that provided bilingual staff to attend transition planning meetings with families. Taken together, these findings suggest opportunities to better support Hispanic/Latine families with autistic children during the kindergarten transition as they may face distinct challenges compared to White caregivers.

Prior studies suggest school personnel may take a deficit-based perspective by discriminating against Hispanic/Latine caregivers and their autistic children, assuming they are not capable due to language barriers, negatively affecting families’ ability to access and receive school-based services (e.g., Burke et al., 2018; 2020). Yosso’s Community Cultural Wealth framework challenges this deficit view of people and communities of color. Yosso’s view shifts the focus from traditional forms of cultural capital that inherently are related to white privilege (such as wealth, income, and social status) to the assets communities of color possess and utilize as cultural capital (Yosso, 2005). Sources of capital include aspirational capital (ability to maintain hope in the face of barriers), familial capital (the communal bonds between family and community and the accumulation of generational knowledge), social capital (utilization of social networks and community resources), linguistic capital (captures the diverse communication styles employed and languages used), resistance capital (skills and knowledge accumulated through experiences of oppositionality and challenging inequality) and navigational capital (practices used to maneuver through institutions that are not intended for communities of color (Yosso, 2005).

The purpose of this study was to investigate the transition to kindergarten experiences of Hispanic/Latine caregivers of autistic children. Specifically, the study aims were to (a) understand challenges in the transition to kindergarten, (b) examine strengths or facilitators caregivers used during their child’s kindergarten transition, and (c) explore how cultural factors may have affected the kindergarten transition experiences.

## Methods

### Participants

Approval for the study was obtained from the University of [university blinded for peer review] Institutional Review Board (IRB). Following IRB approval, participants were recruited from a separate study of caregivers of autistic children who transitioned to kindergarten between 2016 and 2021. After participating in a survey study, an additional survey question asked caregivers if they would be interested in participating in another study (i.e., the current focus group study). If they indicated interest, they were directed to screening questions based on inclusion criteria. Inclusion criteria were that participants must be a caregiver of an autistic child; must speak Spanish, English, or be bilingual; and must identify as Hispanic or Latine. If caregivers met criteria, they were prompted to provide contact information in a separate form so they could be reached to consent for the current study. Participants were contacted via phone and email to schedule an individual Zoom meeting to provide further information about the study and obtain consent. Participants were consented in their identified preferred language (English or Spanish).

Participants included four biological mothers of autistic children. In total, 30 participants met screening criteria and indicated interest in the study. Of those, 23 did not respond to follow up phone calls and emails to participate. Further, although three respondents met inclusion criteria during online screening, upon follow-up, they did not meet inclusion criteria of identifying as Hispanic/Latine. Demographic information and pseudonyms used to identify the four participants, and their children is in Table 1. All participants were scheduled to participate in a focus group provided in English and Spanish. However, due to a scheduling conflict among participants, a separate, single interview using the focus group questions was provided to one participant and the remaining three engaged in the focus group.

**Table 1:** Participant demographics

Participant	Gender	Role	Ethnicity	Language	Education	SES indicator	Generational Status
1 “Wila”	Female	Biological parent	Hispanic/Latine	Bilingual (English & Spanish)	Highschool/GED	Receives SSI/WIC	Second generation
2 “Elena”	Female	Biological parent	Hispanic/Latine	Spanish	Highschool/GED	Child receives free/reduced lunch	First generation
3 “Victoria”	Female	Biological parent	Hispanic/Latine	Bilingual (English & Spanish)	Some college	Receives medicaid	First generation
4 “Malena”	Female	Biological parent	Hispanic/Latine	Bilingual (English & Spanish)	Associate’s degree	Child receives free/reduced lunch	Second generation

Name in quotes represents participant’s pseudonym

### Measures

Participants completed a short demographic questionnaire before the focus group to provide information about their preferred language, race, ethnicity, generational status, and socioeconomic status. The interview protocol was adapted from a focus group topic guide used by Starr et al. (2016) and interview questions used by Smith et al. (2021). These protocols were used for conducting interviews with ethnically and linguistically diverse parents and teachers to examine school transitions for autistic children. Small modifications were made to the questions to align with the current study’s purpose as the questions posed in Smith et al. (2021) were not specific to the kindergarten transition or the experiences of Hispanic/Latine caregivers. Changes including changing the wording in questions such as instead of “Would you describe your transition from early intervention/primary/middle school as successful? Why or why not?” to Would you describe your child’s entry into kindergarten as successful? Why or why not?” to clearly specify the kindergarten transition. Other changes included simplifying language used in the questions. For example, instead of “what was the biggest barrier to the transition?” and “what was the most effective support during the transition” the authors shifted this to “what helped the most to prepare your child for kindergarten?” and “what was the hardest part about your child entering kindergarten?” In addition, the protocol used in Starr et al. (2016) consisted of a topic guide, not interview questions, which was used to inform questions asked in this study. During the focus group, each caregiver was asked seven questions (see Table 2).

**Table 2:** Focus group guiding questions

Question asked
1. Would you describe your child’s entry into kindergarten as successful? Why or why not?
2. What helped the most to prepare your child for kindergarten?
3. What was the hardest part about your child entering kindergarten?
4. Did you have concerns or worries about your child going to kindergarten before they started? Can you tell me about these?
5. Did you feel like there were any language barriers during the process of preparing your child for kindergarten? Can you tell me about these?
6. Were there things that surprised you about what the school expected from you or your child during their entry into kindergarten?
7. What would have been helpful for your child’s school to do to support you and your child when they entered kindergarten?

### Procedures

The focus group and interview were conducted by two Spanish speaking research assistants who served as moderators and translated the interviews. The primary author provided oversight of the focus group and interview process. The primary author and one of the research assistant’s qualifications included being doctoral level graduate students with experiences working with autistic children and their families and in schools and systems that serve families who identify as Latine. The second research assistant’s qualifications included a bachelor’s degree in psychology, extensive experience working on projects with autistic children and their families, specifically those who identify as Latine. Research assistants served as moderators and were trained to obtain consent and facilitate focus groups and interviews. Training included review of the consent and interview protocol and how to respond to participants and ways to facilitate participation. An initial individual meeting was held on Teams to verify participants met criteria for the study and to consent participants. The moderator read the consent form to participants and collected oral and written consent for participation and recording. The focus group and interview were then scheduled and held on a separate date on Microsoft Teams using a semi-structured interview protocol (see Table 2).

The primary author and the research assistants attended both the consent meetings and the interviews. Those primary author introduced herself to participants during the consent process and co-led the focus group with the research assistants. The focus group was conducted in English, at the preference of the participants. The individual interview was conducted in Spanish, and led by a research assistant, with the primary author in attendance. The primary author and two research assistants each asked questions from the interview protocol. Additional probes and/or clarifying questions were provided if question was not directly answered. T. The focus group and interview each lasted 60 min, and participants were compensated with a $50 Walmart Gift Card.

### Data Analysis

For transcription, Microsoft Teams provided audio recordings and transcriptions of the focus group and interview. Each transcript was checked once by one of the research assistants and then checked by the primary author. To ensure confidentiality, participants names were changed to pseudonyms. Participants were contacted regarding their preference of pseudonym, as allocating pseudonyms to participants without their input is not recommended as naming is a personal choice, and assigning a name without participant input could reflect power imbalances, racial/ethnic stereotypes, and not honor participant preferences (Allen & Wiles, 2016; Heaton, 2022). Of the four participants, two chose to self-select a pseudonym, and two preferred for their pseudonym to be selected by the researchers. If participants referred to their child, their names were changed to a general yet neutral term (“my child”) to maintain confidentiality. Next, the transcription from the Spanish interview was translated into English by a bilingual Spanish speaking research assistant, and then back-translated by a different bilingual Spanish-speaking research assistant to ensure accuracy in translation and meaning.

After transcription and translation were complete, a reflexive thematic analysis was conducted following the six steps outlined in Braun and Clarke (2006) and adhering to the suggested procedures in Braun and Clarke (2012, 2022). The first step, “Familiarizing Your Self with The Data” (Braun & Clarke, 2012), consisted of the primary author repeatedly listening to the recordings of the focus group and interview, as well as reading and re-reading the transcripts. The two research assistants also spent time reviewing the transcriptions. Next, in the “Generating Initial Codes” step, the primary author and research assistants read transcripts line by line and initial codes that summarized patterns, thoughts, and ideas that emerged from participant responses were annotated in a column next to the transcript. Then transcripts were chunked by participant responses and coded with each response. They were re-read and coded at least twice, during which codes were added, modified, and refined. This included discussing additional thoughts or refining definitions of codes. Table 3 includes examples of coding during the data analysis process. The transcripts were then reviewed for codes by the two research assistants. The team met to discuss initial impressions and codes, and a list of 35 codes were developed. Next, in step 3 “Searching for Themes”, the list of codes was sorted and grouped into potential themes. The primary author and research assistants met to combine codes to discuss and create potential themes and then to reach consensus among themes. In step 4, “Reviewing Potential Themes”, the themes were reviewed and revised. Then, in step 5, “Defining and Naming Themes”, the names of the themes were revised to ensure they accurately reflected the data and scope of the theme. Themes were revised to avoid overlap and over-generalization. Lastly, in step 6, “Producing the Report”, themes and interpretative analysis of the data were written. The following themes were identified: *Importance of Proactive and Ongoing Partnerships Between Caregivers and Schools, Navigating Unfamiliar Language and Processes, The Need for Dissemination of information about Autism to Teachers*, and *Support from Trusted Systems.* These themes, subthemes, and corresponding codes are listed in Table 4 and codebook is provided in supplementary materials. The primary author and research assistants highlighted excerpts from the data that stood out to them while coding to potentially include in the manuscript. The primary author then reviewed the selected excerpts following the recommendations of Lingard (2019). This included consideration of whether quotes were illustrative, succinct, and representative of the data.

**Table 3:** Data analysis example

Excerpt	Initial coding	Re-coding	Theme
“Umm, I’ll go first. The transition part was pretty easy cause the I don’t know if I mentioned it to you. I was able to get. Our IEP done during the summer.”	Transition support from school (IEP)	Transition practice used by caregiver Transition practices use by school	Importance of Proactive Partnerships Between caregivers and schools
“Now looking back The teacher that that he had, I love that she was very caring and accommodating. But what I didn't know was that she was just coddling him and not empowering his potential.”	Child-teacher relationship Alignment/misalignment of goals Transition support from school	Child-teacher relationship Difference in goals Trust between home and school	Importance of Proactive and Ongoing Partnerships Between caregivers and schools
I would like to add too. I never, no, nobody in my family had had any special needs with, you know, in the school systems here in the US as far as I know. So this was my first time navigating those waters. I did not realize I just assumed that the schools had the child's best interest, and I learned that they really don’t care	Parent knowledge (school systems/special ed) First time navigating school system Alignment/misalignment of goals	Family support Parent knowledge (school systems/special ed Cultural differences Difference in goals	Navigating unfamiliar language and processes

**Table 4:** Themes, subthemes, and corresponding codes

Theme	Subtheme	Codes
Importance of proactive and ongoing partnerships between caregivers and schools	• Supports child adjustment to the transition • Shared interest in advocating for child • Alignment of goals between school and home • Power differentials	• Caregiver-school relationship • Child-teacher relationship • Alignment of goals • Difference in goals • Relationship challenges with school • Transition practices used by school • Transition practices used by school • Trust between home and school
Navigating unfamiliar language and processes	• Too much technical language • Cultural differences in exposure to special ed system • Language barriers • Lack of understanding of IEP processes • Need for parent knowledge of special education	• Communication between home and school • Language barriers • Cultural differences • Discrimination • Difficulty accessing services and support • Caregiver needs
Need for dissemination of information about autism to teachers and the community	• Need for teacher training about Autism • Need for Caregiver and Community training about Autism • Impact of delayed diagnoses on services • Caregiver guilt related to delayed diagnoses	• Child behavior • Inclusion • Exclusion • Caregiver knowledge **or** Caregiver knowledge (lack of) • School/teacher knowledge of **or** School/teacher knowledge (lack of) • Delayed ASD diagnosis • ASD misinformation • Behavioral challenges • Social communication challenges
Support from trusted systems	• Caregivers as advocates • Support from other caregivers • Difficulty accessing support from school • Support from medical and therapy providers • Impact of Covid-19 on support sources	• Advocacy(from caregiver) • Lack of transition support/practices from school • Information from school • Information withheld from school • Community supports • Family support • School resources • Non-school based intervention • Child needs

## Results

### Theme 1: Importance of Proactive and Ongoing Partnerships Between Caregivers and Schools

All participants endorsed the importance of proactivity in initiating the partnerships with the school prior to transition and maintaining communication during the transition process. Participants identified using practices such as getting to visit the school early, meeting the teacher early, having the same teacher as preschool, and holding an individualized education plan (IEP) meeting to discuss the transition process were noted as helpful processes. Wila shared,

*…*From that point we set her up in the IEP so we would have a meeting every year. You know, for her goals and everything. And pretty much she has been pretty close to reaching her goals every year.

When relationships were established early, participants expressed they felt it was easier for their child to acclimate to the school environment and that they were better understood by school staff. When relationships were not established early on, caregivers explained this made it both difficult for them to advocate for their child and hard for their child who was unfamiliar with the school environment and teacher. For example, Malena explained that although her child had an autism diagnosis, the school evaluation process was not done proactively. It was initiated after behavioral challenges emerged during the kindergarten transition, which made the initial transition difficult. Victoria recounted her child’s experience:

So moving forward to a different school was quite difficult for him and he continues to be difficult for him, he went in and had one teacher and had her for a couple of weeks and then they moved them into a different area and all of that, along with everything that was going on, was very traumatic for him and it continues to be traumatic in the 4th grade.

Further, participants highlighted it was not just proactive communication that was important, but that this relationship was ongoing, and the school staff (e.g., teachers, administrators) were continuously dedicated to working with their child and cared about their progress. Malena explained that,

So, I think for my child, that school, the principal, the staff, they were interested in what he was happening, what's happening to him and what he was going through. So, I think that was like the steppingstone to set my child up for where he is now.

What Malena and the other mothers described was not simply, just a relationship, but that ideally, there was a shared partnership with the school that was established early on in her child’s school experience. When there was an ongoing shared interest in the child and shared goals, it seemed more steps were taken to set her child up for success. Wila, whose child’s IEP was established proactively in pre-kindergarten and who had kindergarten staff she was familiar with, found that school staff offered a lot of help and individualized support and understanding of her child. They provided help with adaptive tasks such as toileting and used reinforcers that were motivating and meaningful for her child. Proactive partnerships included shared goal setting and decision making for their child during the transition process and throughout school. Participants expressed a desire to take an active role in the process, with Malena sharing, “now I’m the one that helps set up goals. They don’t do it anymore. I contribute to the goal setting because before he was having speech therapy, and I didn’t see a lot of progress…” Other participants also valued alignment of the school’s goals with their own goals for their child and felt listened to when the goals created for their child at school considered and aligned with their own. Wila acknowledged the importance of this in helping their child:

So for me, pretty much every time we have an IEP meeting they call me ahead of time and tell me what they have noticed at school and if it’s the same things that I've noticed at home and we try to get on the same page that asked what are some of the goals that you want us to, to add or if she needs help still with, you know, with the dressing and little stuff like that, transportation... So, we do try to get on the same page whenever we do the meeting.

Conversely, some participants felt there were adversarial relationships with their child’s school and that their insight, goals, and information about their child did not matter. Specifically, Malena reported that,

One thing I did learn on my last IEP meeting because I've been fighting for OT [occupational therapy]. Is that they talk amongst themselves and say what they feel they need for the child. And then when I come in there and say what I need for my child in the school it is dismissed because everybody else is on the same page. You know that it’s just them holding the key… in any of the IEP meetings, we weren’t equal partners. They made it seem like they're the ones in charge and you're the one listening to their decisions.

This sentiment captures feelings of dismissal and inequality throughout the transition process. It was echoed by others who expressed similar experiences where it did not appear the school teams were taking steps to advance shared goals for their child.

### Theme 2: The Need for Dissemination of Information about Autism to Teachers and the Community

When discussing the kindergarten transition process, all caregivers endorsed how the degree of knowledge about autism and special education influenced their child’s kindergarten experience. Teachers lacking knowledge about autism and misunderstanding the needs of their child were identified as influential factors in the transition process. Teachers’ understanding of their child’s specific sensitivities, abilities, and using strategies such as task analysis and behavior management strategies were identified as qualities that helped strengthen their child’s adjustment to kindergarten. Caregivers in this study also reported that in some cases, teachers did not understand their child’s abilities, had misconceptions about children on the autism spectrum, or were not sure how to manage behaviors. Elena shared that:

Y las maestras creo que en un principio o hasta cierto punto, ellas trataban de trabajar con él. Pero ya después yo creo que, ya como que empezaron a cansarse y pues ya me decían, me sugerían de que lo llevara al doctor para que le dieran medicamento para que pues fuera un poquito más fácil trabajar con él.

[And the teachers I think in the beginning or at least at a certain point, they tried to work with him. But then afterwards I think they started to tire and well they would tell me, they suggested that I take him to the doctor so they could give him medication so that like he would be a little easier to work with.]

Elena’s experience highlights the need for increased training in managing behaviors by school personnel as well as her experience of feeling her child was not “easy” for the school to work with or preferred. For three of the four caregivers, they expressed agreement in not feeling like their child was understood by their teachers. Elena, even reported that, [referring to teachers].

Siento que a veces a los maestros, como que a veces no saben, no sé si decir si ellos tienen el conocimiento o la preparación de cómo trabajar con casos así y a veces ellos como que encapsulan a los niños porque su cerebro trabaja diferente, si pasa algo malo, ya siempre va a ser tu culpa. A veces ellos creo que no tienen la preparación o el entrenamiento de cómo tratar con esos casos así a veces lo que hacen es como poner quejas. Pero más que poner quejas yo creo que es como buscar recursos. ¿De que como ese niño se tiene que educar en base a sus condiciones?

[I feel that sometimes to the teachers, like sometimes they don’t know, I don’t know if I should say, if they have the knowledge/understanding or the preparation on how to work with cases like that. And sometimes they encapsulate kids because their brain works differently. If something bad happens, it will always be their fault. I think that sometimes they don’t have the preparation or the training on how to work with those types of cases and sometimes what they do is like make complaints. But more than make complaints, I think it’s to look to resources. Like how should this child be educated based on his conditions?]

Further, some caregivers felt their child may not have been pushed as much as they were capable of in kindergarten because their teachers were unsure how to best support them. Victoria reflected:

Now looking back. The teacher that that he had, I love that she was very caring and accommodating. But what I didn't know was that she was just coddling him and not empowering his potential.

It appeared that from their experiences, teacher knowledge and understanding of autism not only facilitated the transition but had implications for how their children were viewed and understood by other school staff, as caregivers endorsed instances of teachers not assuming competency, exclusion from general education, and removal from the classroom due to behavioral challenges were used. Further contributing to this, participants also shared they felt there was a lack of knowledge and education about autism for caregivers and within the community. When reflecting on their child’s kindergarten experience, three caregivers expressed similar experiences of being unfamiliar with symptoms of autism and wishing they had known more about it to better support their child. They also shared guilt surrounding their denial of their child’s autism symptoms and reluctance to have them identified. Two caregivers expressed wishing their child would have been diagnosed earlier to permit access services to help prepare for kindergarten. Participants also recalled attributing their child’s challenges to language delays initially, and felt if their child had accessed services earlier, they might have had fewer behavioral, social, and communication difficulties when they entered school. Malena recounted her experience:

I was in the middle of realizing that my son might have autism, he was around 3. And my pediatrician is the one that brought it up. I was in huge denial…

If I could change that, it would be just me accepting what the doctor and my husband were really trying to tell me instead of getting him the help sooner instead of me saying oh, it's a developmental delay.

Additionally, participants asserted that more knowledge about autism is needed in their community, and that both other parents and their own families have misunderstandings about autism. When discussing how knowledge about autism may have helped their child’s kindergarten experiences, three caregivers reported holding personal feelings of denial about their child’s symptoms and that other family and community members also attributed their child symptoms to other delays. Wila said,

In my family I don’t think we have anybody with any special needs or I didn’t even know what autism was. You know, I had heard of autism, but I didn’t know what it was.

Victoria and Wila wondered whether the feelings of denial shared by the group were unique to those of Hispanic/Latine ethnicity, or their community’s culture. Victoria shared that in her church, she feels that her child’s behavior is stigmatized and there are misconceptions about autism, as those in her church community will often ask “what's wrong with him?” She added,

I feel that the whole community, everybody could benefit from understanding that autism is not a disease, it’s not an illness, that it is a different way of viewing life. There’s not enough education, educational material or, you know, out there and it should be. That's the bottom line.

As a result, practices such as exclusion from general education and removal from the classroom due to behavioral challenges were used. Participants reported taking it upon themselves to search for more information by looking online, reading books, and researching terminology used in meetings with their child’s school to support their advocacy efforts. For example, Malena explained with regards to learning about autism, “I learned that summer, I’m still learning, I read books, I do a lot of research online, I participate in SPARK”. Malena’s quote captures the commitment participants felt to expanding their knowledge in this area and contributing to knowledge basis to help others learn.

### Theme 3: Navigating Unfamiliar Language and Processes

Language was an especially important topic to participants. All participants identified as being fluent Spanish-speakers, with three identifying as bilingual (English and Spanish). Despite English proficiency, participants also unanimously agreed that they initially had difficulty understanding parts of the transition process and special education when attending meetings with their child’s school. Specifically, they endorsed feeling that the language the school used to communicate about their child was too technical and confusing. Instead, they wished that school-based professionals would be more patient and provide explanations in simpler language. The mothers in this study shared experiences of feeling overwhelmed and “thrown” into meetings with school staff with limited knowledge and understanding about what was being discussed.

Participants explained the need for laymans terms and simplified language when communicating with their child’s school. In general, all participants identified that difficulties understanding information were hurdles in advocating for their children in school. This was particularly salient for those with limited English proficiency, limited experience with the U.S. school system, and who identified as first generation. For the two caregivers who identified as first-generation immigrants of Hispanic/Latine descent, they described being unfamiliar with the school system in general and special education in the United States and their countries of origin. Malena shared that:

Nobody in my family had had any special needs with, you know, the school systems here In the US as far as I know. So, this was my first time navigating those waters.

Elena discussed her experiences meeting with school staff during her child’s kindergarten transition as monolingual Spanish speaker.

Creo que eso, explicar los términos, como a un lenguaje más cómo decirlo como un poquito, que se entienda más. No, términos tan profesionales porque para el profesional, el si lo va entender verdad, pero para uno de papá creo que más en este nivel que uno apenas va comenzando con ellos. Si es un poquito este, eh, bastante dificil el entender de lo que está hablando.

[ I think it’s that, explaining the terms, in a language more...how do you say…a little bit that is more understandable and to communicate in layman’s terms where people understand what you're saying.]

Elena explained that she sought clarification by communicating by writing down notes and questions, taking advantage of her speaking Spanish by communicating her questions to the interpreter to try and clarify terms. Other participants asked questions, brought written agendas, and looked up unfamiliar terms.

### Theme 4: Support from Trusted Systems

When asked about what they wish they could change about their child’s kindergarten transition, caregivers shared that they wish they would have known how to advocate earlier and that other parents were a helpful resource. Other parents were beneficial in educating them about autism, how to navigate the school system, and the transition process. Trusted sources of information and support seemed particularly important, as information about the transition was withheld or not provided to them. For example, when asked what the biggest challenge in the kindergarten transition was, Malena responded with:

I think it was just them [the school] not giving us all the programs available, not letting us know. Yes, you have a say in this meeting not letting us know what is available to us as parents and to our children.

Participants expressed that information about class options, accommodations, and services to support their child were not clearly communicated. Victoria expressed her disappointment with this relationship:

The school has the school’s best interest and how they can save money or how they can cut corners and throw our children under the rug or, you know, put them in a in a setting where you know they're over there, leave him alone… I was trusting the school that they had their best interest for my child and that he would thrive because that’s why they’re there for.

For Victoria, the actions of her child’s school fostered distrust and consequently, has resulted in her not viewing the school as a source of support or information. Victoria explained that it was through other parents that they learned about their rights regarding the special education process and what they can request from their child’s school to help support them. In addition to talking with other parents, participants cited their child’s therapists and psychologists as resources that were helpful. For example, Malena acknowledged she was initially hesitant to enroll her child in school, but it was her child’s psychologist who evaluated him that encouraged her and shared the potential benefits. Additionally, other participants shared their child’s pediatrician provided information about autism to them. Elena, who expressed feeling shy advocating for her child during school shared that she was more comfortable communicating about school challenges with their speech and occupational therapists, who shared information and strategies that were helpful. When asked where she got information from, Elena shared:

“Solamente se lo platicaba con las terapistas, más que todo con la terapia de habla.” [I would only talk about it with the therapists, more than anything with the speech therapist.]

Participants highlighted navigational capital and the support that schools can offer when positive home school relationships are developed. Malena recounted her child’s experience,

…and that's how we came across Miss R who was the principal at the ELC in [school] district…and through her and the counselor, and her staff, we learned that, hey, this is how we can start approaching IEP meetings. And she educated us. She invested her time in my child and in educating us as parents.

Participants also identified familial capital, as their families and partners were sources of support. During the kindergarten transition, Malena identified that her husband was especially helpful in searching for resources and information to support their child.

## Discussion

Caregivers of autistic children have reported difficulties trying to support their child’s transition to kindergarten (Marsh et al., 2017). Limited research has addressed the specific experiences of Hispanic/Latine caregivers of autistic children, which is warranted as they are more likely to experience communication and language differences, discrimination, and difficulties building relationships with school staff (Burke et al., 2020, 2021). This study provided valuable information by exploring the experiences of for four Hispanic/Latine mothers of autistic children during their child’s kindergarten transition. Themes identified in this study serve to better understand the challenges in the transition to kindergarten, strengths or facilitators caregivers used during their child's kindergarten transition, and the cultural factors that are intertwined with their experiences. Consideration of cultural factors and Yosso’s cultural wealth framework is included in the discussion of findings in the to better understand strengths and types of capital Hispanic and Latine caregivers utilize.

### Barriers and Opportunities for Support Identified During the Kindergarten Transition

The mothers in this study identified challenges and opportunities to further support the kindergarten transition process for their children. The theme *The Importance of Proactive and Ongoing Partnerships Between Caregivers and Schools*, captures the reported challenges in fostering relationships with their child’s school. Such challenges echo prior findings that indicate increased systemic barriers to home-school relationships for Hispanic/Latine families and families of color (Burke et al., 2019; Rios & Burke, 2021). Mothers reported that the factors that influence relationship quality, also influenced knowledge, supports, and services received for their child. This is a challenge highlighted in previous research, as well (e.g., Chen et al., 2020; Starr et al., 2016). This study extends on such prior work by underscoring the importance of proactivity in the transition process by establishing relationships with school systems early (i.e., before the school year), as contact has been suggested to be a facilitator to successful transitions and contributor to home school relationship quality, which can impact transition difficulties, academic success, and social adjustment (Ishikawa et al., 2024; McGhee Hassrick et al., 2021). Participants noted a desire to establish relationships with their child’s school early, but the forms of capital utilized were not consistently received by schools. For some participants their use of navigational and resistant capital to advocate for their child was viewed as adversarial instead of a strength.

The theme *The Need for Dissemination of Information about Autism to Teachers and the Community* emphasizes the overwhelming agreement that more knowledge is needed across systems (e.g., home, school, and community) to facilitate smoother transitions. When participants identified teachers and staff as knowledgeable in this area, information about the kindergarten transition was consistently shared and they appeared to better understand their children’s needs. This is consistent with prior research suggesting gaps in teacher knowledge related to autism (Chen et al., 2020; Fontil et al., 2020). Participants also shared a need and desire for additional information about autism, special education, and the kindergarten transition process. Participants experiences were reflective of misconceptions of autism, disparities and delays in diagnosing autism, which may have contributed to disparities in service access at school, which are common themes experienced by Hispanic/Latine autistic children (Burke et al., 2019). This finding may suggest the of the need for dissemination of information about autism to caregivers and school staff alike. Specifically, highlighted potential opportunities to utilize social and familial cultural capital to expand knowledge in this area to teachers and families in their communities.

Within the theme of *Navigating Unfamiliar Language and Processes*, language barriers and use of technical jargon in transition planning meetings were noted to impede the ability to fully understand plans being implemented and services offered during special education and transition processes, which can strain relationships and communication (Burke et al., 2021; Smith et al., 2021). In this study, caregivers who identified as first-generation immigrants reported additional barriers, as they were not familiar with the U.S. school system or curriculum and needed more information on how to best support their children. Language differences and use of an interpreter without a cultural broker to provide additional cultural context, may contribute to misinformation, misinterpretations, and information not being shared. Such differences may perpetuate the erroneous belief that caregivers are not motivated or able to serve as advocates for their children (Park, 2024). Failure to aknowledge or be open to the community cultural wealth (i.e., knowledge, skills, social connections) and forms of cultural capital caregivers possess when cultural and linguistic differences are presence, may create additional communication barriers and relationship strains on potential support systems (i.e., school personnel) that inhibit the transition process.

For the mothers in this study, trust was an important component of relationships that were supportive. The theme *Support from Trusted System*s reflects the importance of going to trusted systems for support and for three participants, the overall sense of distrust that developed in the relationships with their child’s school. Trust decreased when participants learned from other caregivers or sources of social capital that their child’s school had not provided all the information regarding services or programs that may be available for their child, or felt their child was not well support to succeed. Following these experiences they instead referred to alternative sources of support and cultural capital during the transition process.

### Caregiver Strengths that Facilitated the Kindergarten Transition

Despite challenges, the mothers in the study possessed many strengths and utilized these strengths to support their child’s entry to kindergarten and schooling experiences. This study highlights facilitators to the transition process including the strengths and sources of community wealth and assets of cultural capital that they utilized. While relationships may not have been established as early as desired, the theme *The Importance of Proactive and Ongoing Partnerships Between Caregivers and Schools* captures how participants worked to establish ongoing relationships with their child’s school despite barriers. For example, linguistic capital included initiating frequent contact through notes, phone calls, and emails. Navigational capital was drawn on to continue to work within the school system despite feeling disempowered and not heard. Low feelings of empowerment in navigating school services are not an uncommon experience for Hispanic and Latine caregivers (Burke et al., 2020). However, the mothers in this study utilized aspirational capital by maintaining their hopes and goals for their children even when there were barriers and differences in perceptions of goals. When mothers challenged power imbalances by finding the strength to speak up in meetings, and dedication in determining goals for their child, resistant capital was used.

Although language differences and the use of jargon were identified as challenges, this study and the theme of *Navigating Unfamiliar Language and Processes* represents the linguistic capital was utilized to persist in advocating for their children despite unfamiliar terms and language differences. Participants, took notes, came prepared with questions for meetings, asked questions, and researched unfamiliar terms. These findings highlight the need to not only address language barriers, but to listen to parent preferences and use their strengths in asking questions and sharing their preferences to inform practice changes.

Disparities in knowledge of ASD and special education have been noted among Hispanic and Latine families (Burke et al., 2020). While increased dissemination about ASD and special education were identified as an area of need, the theme *The Need for Dissemination of Information about Autism to Teachers and the Community* shed light on the forms of cultural capital used in expressing their commitment to better understanding autism and their child, and to help others in their community do so. The mothers in this study drew on resistant, linguistic, and social capital to increase their knowledge and understanding of autism, special education, and the kindergarten transition. Participants felt they had a duty to protect, educate, and advocate for their child, and other children within their community by sharing the knowledge about ASD and school systems that they acquired. This is particularly salient within the Hispanic/Latine community as familial capital aligns with the value of *familismo,* which describes an enhanced sense of loyalty, reciprocity, and responsibility to care for family members and family functioning and can serve as a protective factor against stress (Martinez & Turnage, 2022). *Familismo* may have been a facilitator for parent empowerment, self-efficacy, and advocacy practices (Martinez & Turnage, 2022).

Caregivers highlighted specific sources of advocacy and support in the theme *Support from Trusted System*s including the importance of going to trusted systems for support. Participants whose felt the relationship with their child’s school was not trusting, sought alternative supports. They drew on social capital and agreed that it was through talking to other parents or caregivers of autistic children that helped them learn advocacy skills. The importance of referring to trusted providers, family, and friends, highlighted the Hispanic and Latine cultural value of *confianza*, the development of mutual trust and the participants strengths in familial and social capital. For participants, this sense of trust, *confianza*, may be a facilitator in forming positive home school relationships (Morale-Alexander, 2021). Trust is a critical element to the transition as it has been identified as a key factor in collaborative and supportive relationships for families during the kindergarten transition (e.g., McGhee et al., 2021; Nuske et al., 2018).

### Implications for Practice

**Fig. 1:**
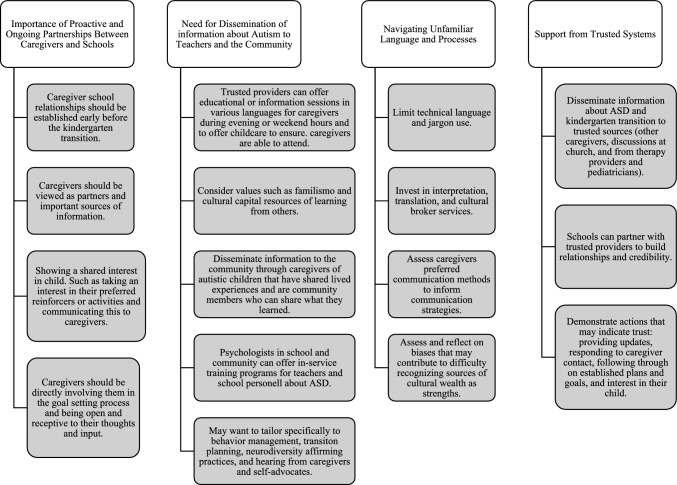
Themes and corresponding practice recommendations

The caregivers’ experiences and themes identified in this study inform several practice suggestions to support future kindergarten transitions for autistic children. Themes and corresponding practice recommendations are presented in Fig. 1. The first practice recommendation is to establish trusting relationships early in the transition process that are ongoing and collaborative. Hispanic and Latine caregivers of autistic children may experience lower levels of empowerment when navigating educational spaces, which is significantly related to quality of the home school relationship (Burke et al., 2020). However, participants in this study also successfully utilized multiple forms of cultural capital. These included working to establish relationships with their child’s school before the kindergarten transition began and practices that facilitated ongoing positive relationships. When participants shared actions from their child’s schools that suggested trust and common goals (e.g., providing specific updates or anecdotes that show a shared interest in their child, following through on established plans and goals, reciprocal interest in their child), participants seemed to view the school as offering more support and help. Therefore, to foster empowerment, this study suggests establishing relationships early, viewing caregivers as important sources of information, directly involving them in the goal setting process, and being open and receptive to their thoughts and input. Discussing a child’s kindergarten entry with a caregiver should include gathering information about culture, parenting practices, understanding of special education, beliefs about autism, concerns about the transition, strengths of their child and their family, and goals for their child. More examples of questions to support planning that may provide information to develop culturally responsive, individualized transition practices are included in Table 5.

**Table 5:** Suggested questions to ask caregivers during the Kindergarten transition

Area	Questions to ask
Caregiver role and family unit	Who lives at home and who helps care for child? (e.g., grandparents, extended family) What is their role in your family? What about in your child’s education planning?
Learning style	How do you understand information best? For example, some people are visual learners or would like others to talk slowly or use handouts
Strengths	What are some family strengths that will help support the kindergarten transition? What are some of your child’s strengths that will help support the kindergarten transition?
Understanding of autism	What does autism mean to you and to your family/community? Where do you get your information about how to support your child? (e.g., community, churches, medical clinics)
Educational system experience	What is your experience with the United States education and special education system? (Have you had any children or community member go through this process that you have talked to?)
Values and goals	Are there any important cultural activities, religious groups, family practices for you and your family? What is important for your family that your child does at school? What are your goals for your child’s transition and schooling? What does your family need to best support the kindergarten transition? What does your child need?
Home based practices	Is there anything you do to support your child that you think would be helpful for us to do at school?
Communication with school	What language do you prefer to communicate and receive communications in? What are the best ways for the school to communicate about your child’s transition with you? (e.g., meetings, papers, phone calls, emails)

Participants indicated their desire for greater dissemination of information about autism and special education. Additionally, participants described a shared sense of responsibility that mothers in this study expressed regarding educating others in their community, which may be conceptualized as *familismo.* The next practice recommendation is that this value and the use of familial capital should be considered in disseminating information and efforts to increase knowledge and awareness. To help facilitate engagement, on-going advocacy, and smooth transitions, *familismo* and familial capital should be considered as assets to Hispanic and Latine families. Perhaps it may be more meaningful and beneficial for information to be offered by caregivers of autistic children that have shared lived experiences and are community members who can share what they learned. Helpful information discussed could include what to expect during the transition process, special education legislation (i.e., 504 plan vs. individualized education plan [IEP]), who to contact, what to ask, and what services are available. While likely an investment of time, resources, and money, it could mitigate the communication and collaboration concerns endorsed by Hispanic and Latine caregivers during the transition process. Participants in this study suggested that they received minimal information from their child’s school about the kindergarten transition and/or special education. Instead, families relied on social and familial capital and obtained information from other trusted caregivers, discussions at church, and from therapy providers and pediatricians to navigate the process. Given this, efforts should be made to draw upon these sources of cultural capital and disseminate information to these trusted sources of support.

To create collaborative, home-school partnerships, school districts would also benefit from adopting this approach and collaborating with communities to disseminate information and holding trainings for teachers specific to neurodiversity affirming practices, strategies to support autistic children in the classroom, and behavior management. Beyond that, given ongoing communication challenges between school staff and caregivers of autistic children, holding panels or round tables for caregivers or self-advocates to talk about their experiences with autism may prove useful in providing differing perspectives. Further, when information was provided by the school, it was not always accessible or easily understood by caregivers. When establishing relationships with students and caregivers school personnel should assess caregivers preferred communication methods, familiarity with school processes, ways to receive communications (e.g., email, phone texts), and preferred method of learning or understanding information (e.g., visual learner vs auditory) as this can inform more effective ways to communicate and support families and children. School staff will want to take care to limit jargon and technical language, provide adequate interpreter and translation services, and use cultural brokers if feasible. School staff are encouraged to reflect on biases that may contribute to difficulty recognizing sources of cultural wealth that caregivers may use to assist in this process (e.g., bringing trusted family to IEP meetings, taking notes and bringing questions, asking questions or for clarification) as strengths and viewing them as adversarial or challenging.

### Implications for Research

Future research should replicate and expand this study and other work to identify opportunities to best support young autistic children and their families during the transition to kindergarten. Research should be aimed at identifying avenues to increase service provision and disseminate information about autism and the kindergarten transition within the Hispanic and Latine community. Research with families from underrepresented and/or marginalized communities often takes a deficit-based instead of strength-based approach, yet this study highlighted strengths in self-efficacy and advocacy that mothers exhibited during the kindergarten transition and beyond for their autistic children. Future studies should systematically integrate community cultural wealth (Yosso, 2005) to further explore transition to kindergarten practices specific to Hispanic/Latine parents or other caregivers (e.g., grandparents) of autistic children and to examine attributes that effective sources of transition support possess. This information could be used to implement interventions to bridge the school and home settings, which would aid in capitalizing on their unique strengths. While this study explores caregiver perspectives, future studies that explore the perspectives of school personnel may be beneficial in understanding areas of need during the transition process.

Studies assessing teacher knowledge and perspectives about ASD are indicated, as well as implementation of interventions to increase teacher knowledge in this area to support effective kindergarten transitions.

Lastly, larger scale studies examining the experiences and perspectives of other SCLD caregivers and families of autistic children are needed since people of color and those from low socioeconomic communities are underrepresented in autism research (West et al., 2016). Concerted efforts should be made by researchers to increase recruitment and retention strategies for minoritized participants into research studies and to focus on engaging in community partnerships such as community-based participatory research, participatory action research (PAR), and community-academic partnerships to foster relationships with communities allowing researchers to reduce and eliminate barriers to participation and conduct research that is relevant, respectful, and supportive of communities (Maye et al., 2021). Additionally, asking families and communities their preferences for research recruitment methods is strongly recommended as is including information in manuscripts about effective recruitment and retention strategies to guide future researchers (Mire et al., 2023).

### Limitations

Results of this study should be interpreted in the context of several limitations.

Participants of this study self-selected; therefore, the participants’ views and perspectives may be influenced by parents’ experiences being either very positive or very negative, and therefore not representative of all views. Moreover, the focus group was conducted by the primary investigator whose race and ethnicity are incongruent with that of the participants in the focus group. This is important to note, given that participants in focus groups that are homogonous ethnicity and race are more likely to have discussions about the influence of culture, race, and ethnicity (Greenwood et al., 2014). However, the focus group was co-moderated by research assistants who were congruent in ethnicity with participants and there was ethnic congruence in the interview conducted in Spanish. Therefore, this limitation may be overcome as the group was co-moderated by individuals who were homogenous in ethnicity, participants were homogenous in ethnicity and had other characteristics in common such as language spoken and being caregivers for autistic children.

## Conclusion

The information and themes identified from this study provide an in-depth look at the kindergarten transition experiences of four Hispanic and Latine mothers of autistic children. Examining their experiences contributed to better understanding challenges that Hispanic and Latine caregivers and children may experience during the transition period. This study also highlighted the community cultural wealth and cultural capital participants brought forward as strengths. The strengths and forms of cultural capital mothers utilized in this study can inform future efforts to promote positive kindergarten transitions. This study provides a foundation for future work to support Hispanic and Latine families and autistic children during the kindergarten transition through developing culturally responsive transition practices.
